# Pemphigus foliaceus as a differential diagnosis in vesicobullous lesions

**DOI:** 10.1590/S1679-45082017RC3828

**Published:** 2017

**Authors:** Louise de Almeida Ferreira Fonseca, Célia Antônia Xavier de Moraes Alves, Ivan Aprahamian, Clóvis Antônio Lopes Pinto

**Affiliations:** 1 Faculdade de Medicina de Jundiaí, Jundiaí, SP, Brazil.

**Keywords:** Pemphigus, Skin diseases, vesiculobullous, Case reports

## Abstract

Given the challenge of clinical diagnosis of bullous skin lesions, this report aimed to discuss the histological changes, the presentation and clinical reasoning for diagnosis of these lesions. At the same time, the importance of the pathology was reviewed to identify these clinical scenarios. In this case report, we highlighted the clinical progression of a case of pemphigus foliaceus.

## RESUMO

Considerando o desafio do diagnóstico clínico de lesões cutâneas de apresentação bolhosa, o presente trabalho procurou discutir as alterações histológicas, a apresentação e o raciocínio clínico para o diagnóstico de tais lesões. Paralelamente, a importância da patologia foi revisada na identificação destes quadros. Neste relato de caso, destaca-se a evolução clínica de um quadro de pênfigo foliáceo.

## INTRODUCTION

The differential diagnosis of vesiculobullous diseases can be considered a challenge in the daily clinic of internal medicine and dermatology. The lesions present an acute onset, and several mechanisms may be responsible for their formation.

Blisters occur at different skin levels. Initially, in the histological, immuno-histological and electron microscopy evaluations, the goal is to find the plane in which the loss of cell adhesion occurs.^1^ Histological diagnosis becomes mandatory to complement the investigation,^2^ although, only the histologic assessment and pathogenic mechanisms do not necessarily assure a correct diagnosis. Therefore, immunofluorescence techniques are a valuable supplement.^3^

Pemphigus foliaceus (PF) is a chronic autoimmune bullous skin disease, which is histopathologically characterized by the formation of intra-epidermal bulla with acantholysis, and immunologically by circulating autoantibodies to the epidermis, which are responsible for the cutaneous lesions.

## CASE REPORT

A 78-years old Caucasian male, born and raised in the urban areas of Jundiaí, State of São Paulo, Brazil, presented “burst wounds in the body” for 20 days. He referred that approximately during 2 months, expressive pruritic lesions appeared on the scalp associated with local burning (burning sensation) with craniocaudal progression. He also reported that the lesions progressed to cervical, trunk, abdomen and underarms areas involvement for approximately 20 days. He had used zync oxide ointment and bath with *Stryphnodendron barbatiman*, which are popular generic treatments, with worsening of the lesions. He had no previous skin lesions, and denied any mucosal involvement or other clinical complaints.

Previously he had hypertension, type 2 *diabetes mellitus*, and obesity. He denied any family history of skin lesions. His current prescription consists in omeprazole, losartan, simvastatin, aspirin and subcutaneous NPH insulin.

Dermatological examination revealed erythematous and bullous lesions, with thick crusts on the surface. Lesions were distributed in scalp, trunk, back, abdomen and underarms. A few bullous lesions on the back presented with flaccid bulla. Nikolsky sign (slight rubbing of the skin results in exfoliation of the outermost layer) was clearly positive. After this finding, dermatologists made the clinical diagnosis of PF. Treatment was started with prednisone 1mg/kg per day associated with antibiotic therapy (initially with cephalexin and later teicoplanin to control a secondary nosocomial infection), topical corticosteroid therapy with hydrocortisone and an immunosuppressant corticosteroid-sparing agent (azathioprine). After hospital discharge, histologically examination of biopsied lesions confirmed PF.

This patient is currently followed as an outpatient and still presents partial remission, with dependence on corticosteroid therapy at lower doses, after several months.

**Figure 1:**
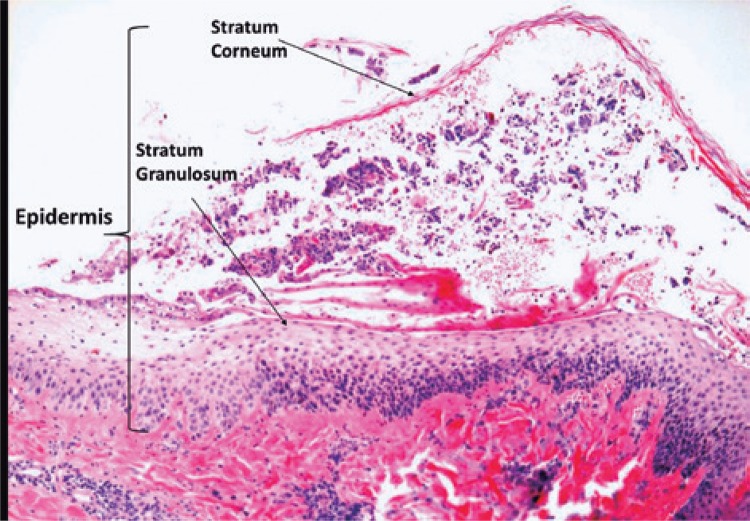
Subcorneal bullous

**Figure 2:**
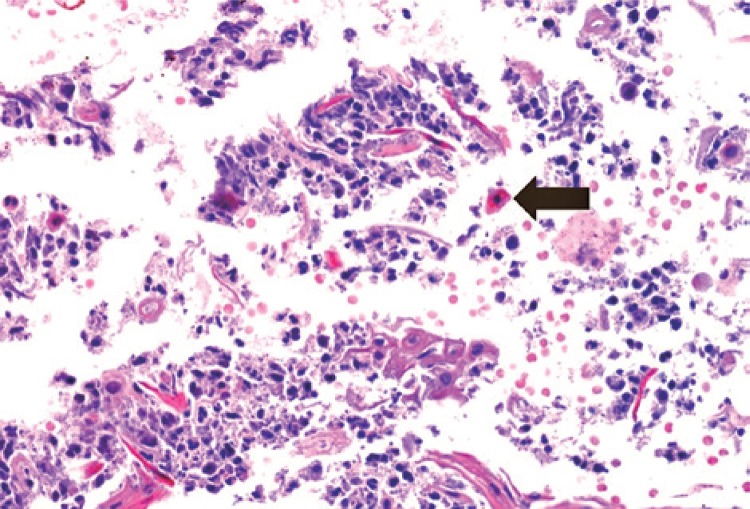
Dyskeratosis/apoptotic keratinocytes (haematoxylin and eosin, magnification 200X)

Histological analysis showed a subcorneal lesion with inflammation (Figure 1) and apoptotic keratinocytes (Figure 2), characteristic of PF.

## DISCUSSION

The patient had a recent history of itching with burning sensation, first in the scalp region, which had a fast craniocaudal progression to the neck, back, abdomen, and underarms. On examination, he presented with flaccid vesicular and bullous lesions with areas of ulceration and thick crusts. The positive Nikolsky sign suggests an intra-epithelial cleavage.

**Figure 3:**
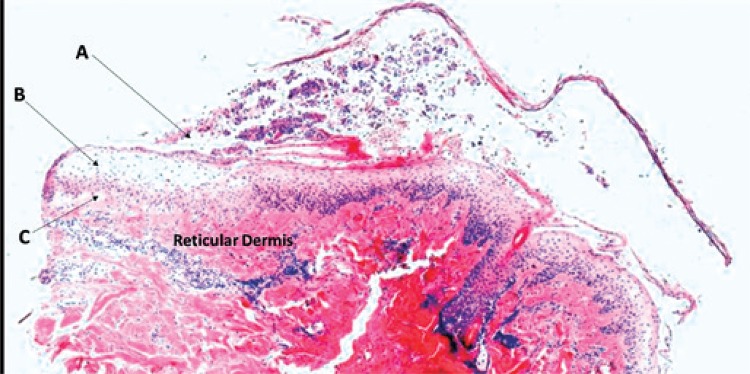
Possible regions of lesions (haematoxylin and eosin, magnification 40X): (A) sub-stratum corneum, (B) suprabasal and (C) subepidermal

Due to the frequently heterogeneous clinical features, it is often not possible to make an exact diagnosis of bullous dermatoses solely on the basis of the clinical findings.^4^ Initially, in the histological and immuno-histological evaluations, and even in electron microscopy, the goal is to find the plane in which the loss of cell adhesion occurs. From the identification of this region, we have some possible pathologies: sub-stratum corneum (represented by PF, impetigo, pustular psoriasis and neonatal toxic erythema), suprabasal (spongiotic dermatitis, Darier’s disease, Hailey-Hailey’s disease, multiform erythema and pemphigus vulgaris), subepidermal (porphyria cutanea, epidermolysis bullosa acquisita, vesicular lichen, bullous pemphigoid, dermatitis herpetiformis, linear IgA bullous dermatosis) (Figure 3).

Once the cleavage site is identified, the mechanism responsible for the lesion is investigated. A histologic finding of intraepidermal bullae with the presence of acantholysis is the main histopathological feature of autoimmune intraepidermal diseases from the pemphigus group.^5^ The autoimmune intraepidermal bullous diseases include defined entities from the pemphigus family: pemphigus vulgaris, PF, pemphigus herpetiformis, IgA pemphigus, paraneoplastic pemphigus and drug-induced pemphigus.^5,6^ In pemphigus vulgaris, there are intraepidermal, suprabasal acantholysis, loss of keratinocytes adhesion, neutrophilic and eosinophilic granulocytes, and a scant perivascular round-cell infiltrate is found in the upper dermal vascular plexus. Pemphigus foliaceus presents a superficial subcorneal acantholysis in the upper stratum spinosum or granulosum and a slight inflammatory infiltration of the upper dermis. In paraneoplastic pemphigus, interface dermatitis with vacuolar degeneration of basal keratinocytes and keratinocytes necrosis with slight acantholysis are seen histologically in association of a lichenoid infiltration in the vicinity of the dermo-epidermal junction zone. IgA pemphigus exhibits an intraepidermal or subcorneal infiltration of neutrophils and classical signs of acantholysis are usually missing.^6^ In pemphigus herpetiformis, acantholytic cells are usually located subcorneallly, and eosinophilic spongiosis is most typical for this variant (Chart 1).

**Chart 1:** Histology and antigen pattern of different types of pemphigus

Pemphigus diagnosis	Histology	Autoantigen
Pemphigus vulgaris	Intraepidermal suprabasal acantholysis	Dsg 3
	Tomb stone appearance	Dsg 1
Pemphigus foliaceus	Superficial subcorneal acantholysis	Dsg 1
	Dyskeratosis	
Pemphigus herpetiformis	Subcorneal acantholysis	Dsg 1
		Dsg 3
		Dsc 1
		Dsc 3
		178kDa protein
IgA pemphigus	Intraepidermal infiltration by neutrophilis or subcorneal infiltration by neutrophilis usually without classical acantholysis	Dsg 1 (IgA)
		Dsg 3 (IgA)
		Dsc (IgA)
Drug-induced pemphigus	Suprabasal acantholysis	Dsg 1
		Dsg 3
Paraneoplastic pemphigus	Interface dermatitis, lichenoid infiltrate of the dermo-epidermal junction, keratinocyte necrosis	Envoplakin
		Pariplakin (IgA, IgG)

Dsg: desmoglein; Dsc: desmocollin; Ig: immunoglobulin.

The major pemphigus sub-types are pemphigus vulgaris and PF. In this disease, IgG autoantibodies (desmoglein-1 and/or 3) break the connection between desmosomes, causing acantolisis.^7^ The pathophysiological process is controversial, but it is certain that pemphigus vulgaris causes mucosal and skin lesions, reaching the suprabasal layer by producing desmoglein-3. Pemphigus foliaceus only causes skin lesions, reaching the subcorneal layer by production of desmoglein-1.

*Fogo selvagem* or “wild fire”, is an endemic form of PF in rural regions of Brazil, and appears to be triggered by an environmental agent, not yet identified.^8^

Direct immuno-fluorescence is used to determine the type of immunoglobulin (IgG, IgA and IgM), and complement fraction (C3, C5b-9) or fibrinogen may be necessary to identify the underlying pathophysiology and its distribution pattern on the basis of the vesicle or bubble in the epidermis, basal linear, or granular membrane. In the case of PF, an intercellular deposition of IgG is observed. Direct immuno-fluorescence is similar between pemphigus vulgaris and PF, but once they have been differentiated by histopathology, direct immuno-fluorescence findings are suggestive of PF up to 100%.^9,10^

It is important to emphasize this is a severe illness and potentially fatal. The diagnosis needs to be quick and the treatment is based on immunosuppression with steroids (topical and systemic), corticosteroid-sparing immunosupressants, immunoglobulins, and anti CD-20.^7^
